# Supplementary material for: Single-trial modeling separates multiple overlapping prediction errors during reward processing in human EEG
Source: Commun Biol. 2021 Jul 23;4:910. doi: 10.1038/s42003-021-02426-1 (PMC8302587; doi:10.1038/s42003-021-02426-1)
Supplement: Supplementary file 1 — Reporting Summary [file 42003_2021_2426_MOESM1_ESM.pdf]

nature research

Corresponding author(s): Colin W. Hoy

Last updated by author(s): Jun 21, 2021

Reporting Summary

Nature Research wishes to improve the reproducibility of the work that we publish. This form provides structure for consistency and transparency in reporting. For further information on Nature Research policies, see our Editorial Policies and the Editorial Policy Checklist.

Statistics

For all statistical analyses, confirm that the following items are present in the figure legend, table legend, main text, or Methods section.

n/a	Confirmed	
☐	☒	The exact sample size (n) for each experimental group/condition, given as a discrete number and unit of measurement
☐	☒	A statement on whether measurements were taken from distinct samples or whether the same sample was measured repeatedly
☐	☒	The statistical test(s) used AND whether they are one- or two-sided<br>*Only common tests should be described solely by name; describe more complex techniques in the Methods section.*
☐	☒	A description of all covariates tested
☐	☒	A description of any assumptions or corrections, such as tests of normality and adjustment for multiple comparisons
☐	☒	A full description of the statistical parameters including central tendency (e.g. means) or other basic estimates (e.g. regression coefficient) AND variation (e.g. standard deviation) or associated estimates of uncertainty (e.g. confidence intervals)
☐	☒	For null hypothesis testing, the test statistic (e.g. F, t, r) with confidence intervals, effect sizes, degrees of freedom and P value noted<br>*Give P values as exact values whenever suitable.*
☒	☐	For Bayesian analysis, information on the choice of priors and Markov chain Monte Carlo settings
☐	☒	For hierarchical and complex designs, identification of the appropriate level for tests and full reporting of outcomes
☐	☒	Estimates of effect sizes (e.g. Cohen's d, Pearson's r), indicating how they were calculated

Our web collection on statistics for biologists contains articles on many of the points above.

Software and code

Policy information about availability of computer code

Data collection	Experimental task paradigm coded in PsychoPy v1.85.3 and run on Windows XP; EEG data collected on the same computer and OS using BioSemi recording software.
Data analysis	Custom Python and MATLAB code used for preprocessing and analysis is available as a GitHub repository (https://github.com/hoycw/PRJ_Error_eeg), which includes system requirements and dependencies.

For manuscripts utilizing custom algorithms or software that are central to the research but not yet described in published literature, software must be made available to editors and reviewers. We strongly encourage code deposition in a community repository (e.g. GitHub). See the Nature Research guidelines for submitting code & software for further information.

Data

Policy information about availability of data

All manuscripts must include a data availability statement. This statement should provide the following information, where applicable:

- Accession codes, unique identifiers, or web links for publicly available datasets
- A list of figures that have associated raw data
- A description of any restrictions on data availability

The datasets generated and/or analyzed during the current study are available in the Open Science Foundation repository and can be found at https://doi.org/10.17605/OSF.IO/JGXFR (ref. 113).

April 2020

Field-specific reporting

Please select the one below that is the best fit for your research. If you are not sure, read the appropriate sections before making your selection.

☐ Life sciences ☒ Behavioural & social sciences ☐ Ecological, evolutionary & environmental sciences

For a reference copy of the document with all sections, see nature.com/documents/nr-reporting-summary-flat.pdf

Behavioural & social sciences study design

All studies must disclose on these points even when the disclosure is negative.

Study description	This study collected quantitative behavioral data from all and EEG data from some participants. Quantitative post-experiment rating data was also collected from a subset of EEG participants (see Supplementary Methods). Behavioral rating data was collected from a separate cohort of participants.
Research sample	EEG data was collected from 41 adult healthy participants (mean ± SD [range]: 20.5 ± 1.4 [18-25] years old; 28 women; 37 right-handed) at the University of California, Berkeley. A separate cohort of 24 healthy adults (mean ± SD [range]: 30.6 ± 5.3 [21-44] years old; 12 women; 22 right-handed) completed an additional behavioral rating study. All subjects reported no history of psychiatric or neurological disorders and had normal, or corrected-to-normal, vision.
Sampling strategy	Data was sought from ~30 participants to exceed the standard sample size in EEG research experiments. Furthermore, the experiment was designed to collect large numbers of trials in each participant for rare conditions (e.g., 600 trials per participant yields 72 trials for rare neutral outcomes on 12% of trials), thereby providing unusually large statistical power for an EEG study even after allowing for artifact rejection. For the behavioral rating experiment, ~20 participants were sought to confirm the findings from the EEG cohort.
Data collection	EEG data was collected by C.W.H., S.C.S., and two undergraduate research assistants (see Acknowledgments) using desktop computers and EEG recording equipment. Participants completed the task isolated in a closed, dimly lit recording booth with minimal visual and auditory distractions. Experimenters but not participants understood the general aims of the study. The behavioral rating experiment data was collected remotely, with participants downloading and performing the task at home and uploading a behavioral log file.
Timing	Data collection began in October 2018 and finished in February 2020. Initial EEG cohort was collected by August 2019, and replication EEG cohort data collection began in October 2019. The behavioral rating experiment was collected from January to March 2021.
Data exclusions	EEG participants were excluded because of technical recording errors (n = 4 datasets with missing EEG or metadata necessary for analysis), excessively noisy data (n = 2 datasets with >3 standard deviation outliers in number of epochs or time points rejected based on visual identification of large, global artifacts), or poor behavioral performance (n = 3 datasets where RT outlier exclusion criteria resulted in <20 trials in any condition), leaving 32 participants for analysis. EEG channel and trial rejection were completed prior to and were therefore blind to analysis results. Behavioral data were rejected based on reaction time cut offs determined a priori by design and analysis constraints (i.e., long RTs too close to baseline periods preceding feedback epochs of interest). Participants were excluded if these criteria resulted in less than 20 trials in any condition. For the behavioral ratings task, participants with prior knowledge of outcome probabilities in the task (n = 2) were excluded after preliminary analyses revealed qualitatively different (less variable) ratings.
Non-participation	No participants dropped out of this study.
Randomization	Participants were not allocated into groups in this study design.

Reporting for specific materials, systems and methods

We require information from authors about some types of materials, experimental systems and methods used in many studies. Here, indicate whether each material, system or method listed is relevant to your study. If you are not sure if a list item applies to your research, read the appropriate section before selecting a response.

Materials & experimental systems

n/a	Involved in the study	
☒	☐	Antibodies
☒	☐	Eukaryotic cell lines
☒	☐	Palaeontology and archaeology
☒	☐	Animals and other organisms
☐	☒	Human research participants
☒	☐	Clinical data
☒	☐	Dual use research of concern

Methods

n/a	Involved in the study	
☒	☐	ChIP-seq
☒	☐	Flow cytometry
☒	☐	MRI-based neuroimaging

Human research participants

Policy information about studies involving human research participants

Population characteristics	See above.
Recruitment	EEG participants were recruited by word of mouth through undergraduate research assistants and either financially compensated or given course credit. Remote behavioral participants were recruited via email list serv and financially compensated.
Ethics oversight	University of California, Berkeley Committees on Human Research

Note that full information on the approval of the study protocol must also be provided in the manuscript.
