# Supplementary material for: Single-trial modeling separates multiple overlapping prediction errors during reward processing in human EEG
Source: Commun Biol. 2021 Jul 23;4:910. doi: 10.1038/s42003-021-02426-1 (PMC8302587; doi:10.1038/s42003-021-02426-1)
Supplement: Supplementary file 2 — Supplementary Information [file 42003_2021_2426_MOESM2_ESM.pdf]

SUPPLEMENTARY INFORMATION:

Single-trial modeling separates multiple overlapping prediction errors during reward processing in human EEG

Colin W. Hoy, Sheila C. Steiner, Robert T. Knight

Supplementary Note 1: FRN peak latency shifts with RPE valence 2

Supplementary Note 2: ERP PE sequence results are robust to biases in subjective reward expectations 4

Supplementary Figures 6

Supplementary Tables 18

SUPPLEMENTARY NOTE 1: FRN peak latency shifts with RPE valence

Examination of the grand-average FRN waveshapes in Supplementary Figure 9a reveals shifts in the latency of the negativity, with losses peaking earlier than wins. Interestingly, neutral outcomes with identical reward values have earlier peak latencies in easy than hard blocks (see inset in Sup. Fig. 9a for direct comparison), despite explicit instructions stating neutral feedback does not reflect performance. Moreover, subjective ratings from post-experiment surveys confirmed EEG participants had explicit neutral feelings towards these outcomes in both easy (mean ± SD on zero-centered 9-point Likert scale: -0.2 ± 1.3; $t(23) = -0.53$, $p = 0.60$) and hard (-0.4 ± 1.6; $t(23) = -1.00$, $p = 0.33$) conditions. However, in our behavioral RL model, neutral outcomes had opposite valence based on contextual expectations, with negative RPE valence for omission of expected wins in easy blocks and positive RPE valence for omission of expected losses in hard blocks.

To determine whether FRN timing shifted systematically according to our RL model predictors, we applied the same multiple regression framework to predict FRN peak latencies. In a general linear model, the only model features predictive of FRN peak latency were RPE value ($\beta = 0.008$, $q_{FDR} = 2.80 * 10^{-8}$; Sup. Fig. 9b) and expected value ($\beta = 0.004$, $q_{FDR} = 0.047$), which are the two predictors encoding valence. A paired samples t test confirmed that FRN peak latencies following neutral feedback were significantly different between easy (mean ± SD: 228.3 ± 0.02 ms) and hard conditions (247.0 ± 0.02 ms; $t(27) = -3.70$, $p = 0.001$), consistent with different reward expectations for easy and hard conditions leading to negative and positive RPEs, respectively, in these

otherwise identical outcomes. This dissociation between subjective ratings and neural signatures of valence suggests our approach can index implicit brain states not revealed by explicit participant report.

SUPPLEMENTARY NOTE 2: ERP PE sequence results are robust to biases in subjective reward expectations

To determine whether subjective reward expectations deviate from those derived from our RL model or impact our EEG results, we conducted an additional behavioral Target Time experiment in which we asked 22 participants to rate their subjective probability of winning using a slider bar after responding but before feedback (see Sup. Fig. 10a for example participant behavior). Comparing these subjective ratings to those derived from our behavioral modeling revealed strong correspondence overall ($r = 0.705$, $p < 10^{-10}$; Sup. Fig. 10b), but this relationship was likely driven by the large difference between easy and hard conditions, as the correlation was significant only within hard conditions ($r = 0.123$, $p < 10^{-6}$) and not within easy conditions ($r = 0.007$, $p = 0.79$). Nonetheless, participants rated their probability of winning significantly higher before wins than losses overall ($t(3156) = -4.27$, $p < 10^{-4}$), as well as within only easy ($t(1575) = -3.52$, $p < 10^{-3}$) and only hard conditions ($t(1579) = -4.29$, $p < 10^{-4}$). These data suggest that despite variance in subjective ratings, participants are sensitive to both the experimental manipulation of difficulty and their own behavior which combine to form reward expectations in our task.

The superior performance of our RL model over outcome-based models and the outcome valence model over the outcome value model demonstrate the importance of incorporating reward expectations when evaluating EEG signatures of reward feedback. However, the rating data from our behavioral experiment indicate participants' subjective expectations were biased relative to our behavioral model such that they overestimated their likelihood of winning in hard conditions and underestimated it in easy conditions

(Sup. Fig. 10c). The biases measured in our behavioral experiment are small on average (mean ± SD in hard: -8.1 ± 10.2%; mean ± SD in easy: 6.3 ± 11.5%), but the results of our regression analyses could be affected by subjective biases in our sample of EEG participants. To address this potential confound, we simulated the effect of adding similar biases to the expected value predictor in our model. Due to the opposite directionality of bias in easy and hard conditions, adding the most extreme bias observed in our behavioral cohort (+/-25% shift in win probability) equalized reward expectations across easy and hard conditions and effectively reproduced the outcome value model tested above (compare model predictions in Sup. Fig. 1c to those in Sup. Fig. 1b). Since the optimistic bias in the hard condition was slightly larger and matches an overoptimistic bias reported in previous studies [70], we also simulated a model with +25% shift in win probability for only hard conditions (Sup. Fig. 1d). Repeating our multiple regression analyses at Fz and Pz with the RL model including subjective bias in hard conditions reproduced all of the main RPE value, RPE magnitude, and probability results from Fig. 2 (Sup. Fig. 4k and 4l). Overall, these analyses show that the level of subjective bias in reward expectations in our task do not affect our results and conclusions.

SUPPLEMENTARY FIGURES:

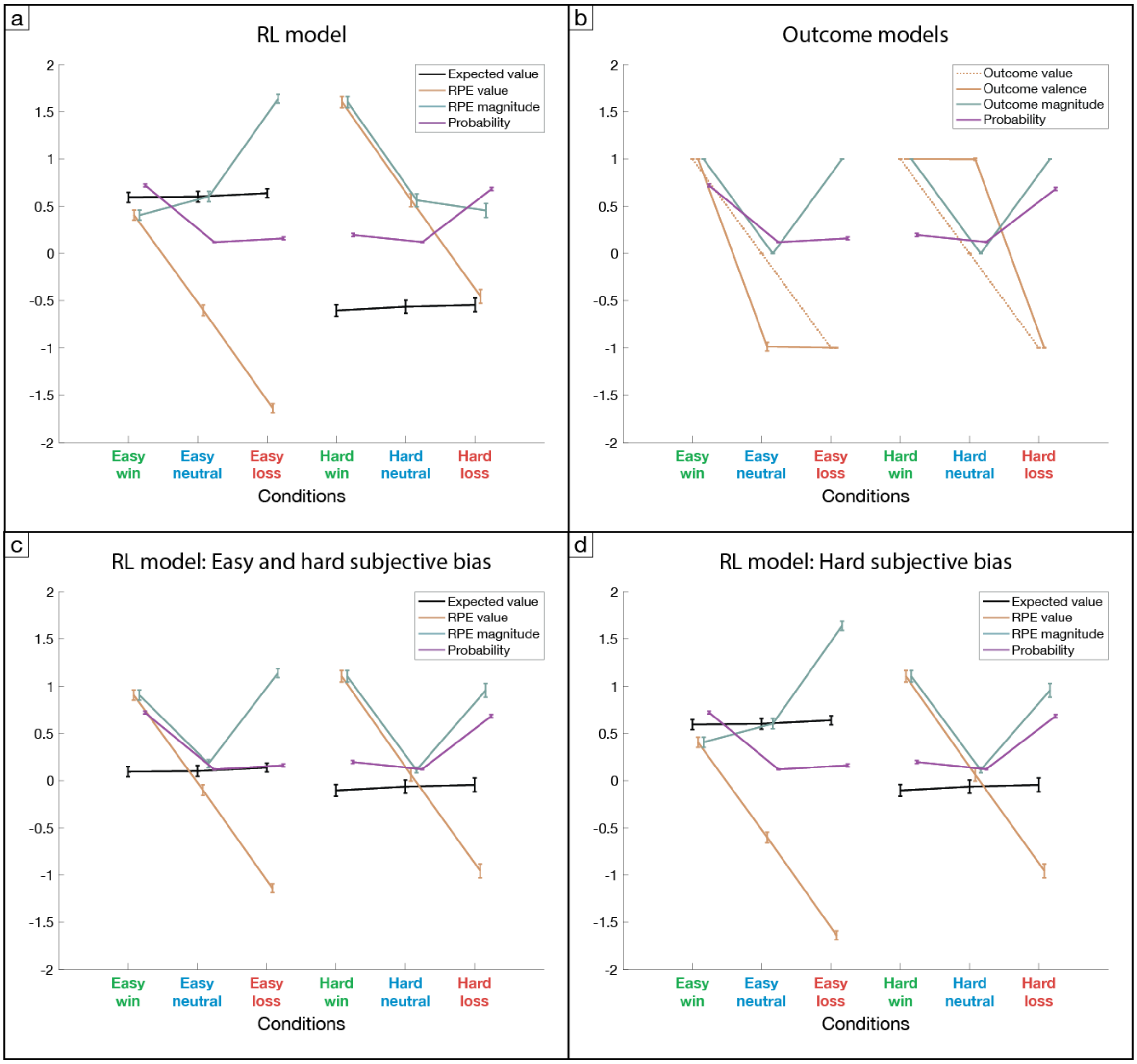

Supplementary Figure 1: Predictions for RL-based and outcome-based models. Error bars indicate standard deviation between 32 participants. (a) Full RL model predictions by condition. (b) Outcome-based model predictors. Outcome value model includes value, magnitude, and probability, while outcome valence model is the same but replaces value with valence. (c) RL model with bias added to expected value in hard (+25% win probability) and easy (-25% win probability) conditions to match most extreme bias found in subjective rating data. Note that this effectively eliminates the difference in

expected value across easy and hard conditions, resulting in predictors very similar to the outcome value model. (d) RL model with positive bias (+25% win probability) added to expected value in hard conditions to match most extreme bias found in subjective rating data.

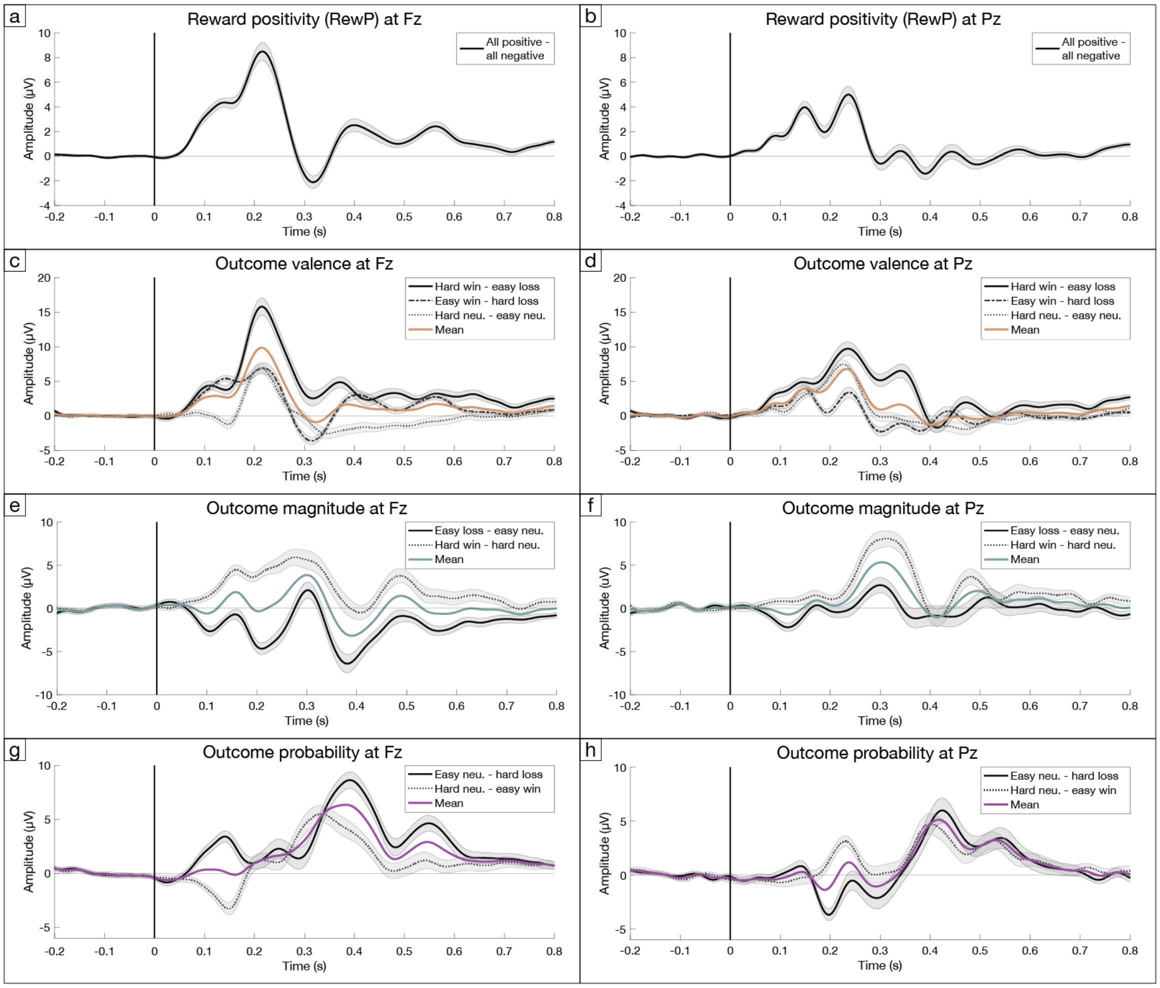

Supplementary Figure 2: Grand-average ERP difference waves to assess outcome valence, magnitude, and probability. Shaded error bars indicating standard error of the mean across 32 participants. (a) Grand-average ERP difference wave at Fz between all conditions with positive RPE valence and all conditions with negative RPE valence, a

contrast commonly used to derive the RewP. (b) Same at Pz. (c) Grand-average ERP difference wave at Fz to assess RPE valence by contrasting pairs of conditions matched for RPE magnitude and outcome probability. Tan line shows mean of these difference waves for comparison with RPE value model coefficients in Fig. 2. (d) Same at Pz. (e) Grand-average ERP difference wave at Fz to assess outcome magnitude by contrasting pairs of conditions matched for RPE valence and outcome probability. Teal line shows mean of these difference waves for comparison with RPE magnitude model coefficients in Fig. 2. (f) Same at Pz. (g) Grand-average ERP difference wave at Fz to assess outcome probability by contrasting pairs of conditions matched for RPE valence and magnitude. Purple line shows mean of these difference waves for comparison with probability model coefficients in Fig. 2. (h) Same at Pz.

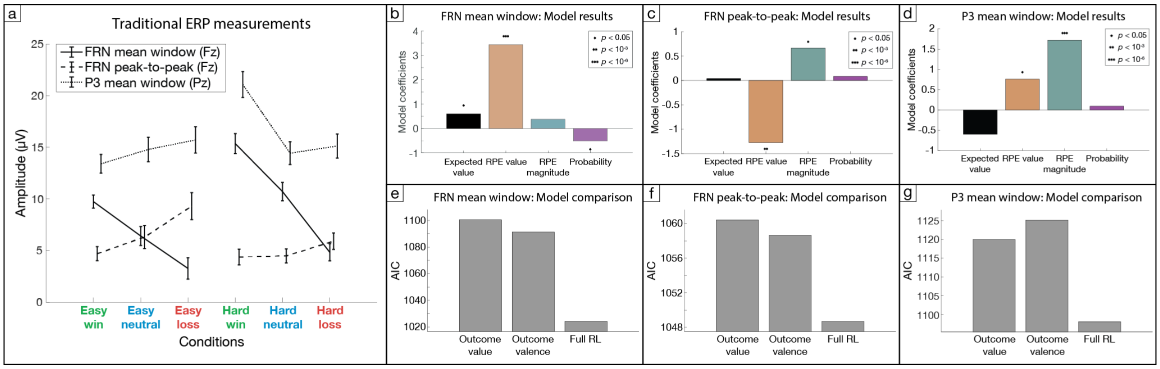

Supplementary Figure 3: Traditional mean window and peak-to-peak metrics support single-trial modeling results but cannot disambiguate individual predictors. (a) Traditional ERP measurements per condition. Error bars indicate standard error of the mean across 32 participants. FRN and P3 mean window estimates are averaged over 100 ms windows centered on the peak latency of the FRN at Fz and P3 at Pz in the grand-

average ERP across all conditions as illustrated in Fig. 2a and Fig. 2b, respectively. FRN peak-to-peak is calculated as differences between amplitudes at FRN and preceding P2 positivity peaks for each participant. (b) RL model coefficients from multiple regression of FRN mean window estimates show significance for valenced expected value and RPE value, and also non-valenced probability ($q_{FDR} < 0.05$; n = 32 participants). (c) RL model coefficients from multiple regression of FRN peak-to-peak estimates are significant ($q_{FDR} < 0.05$; n = 32 participants) for valenced RPE value and non-valenced RPE magnitude. (d) RL model coefficients from multiple regression of P3 mean window estimates show significance for valenced RPE value and non-valenced RPE magnitude ($q_{FDR} < 0.05$; n = 32 participants). (e) Model comparison for FRN mean window estimates shows RL model outperforms outcome-based models that fail to account for expectations. (f) Same for FRN peak-to-peak. (g) Same for P3 mean window analysis.

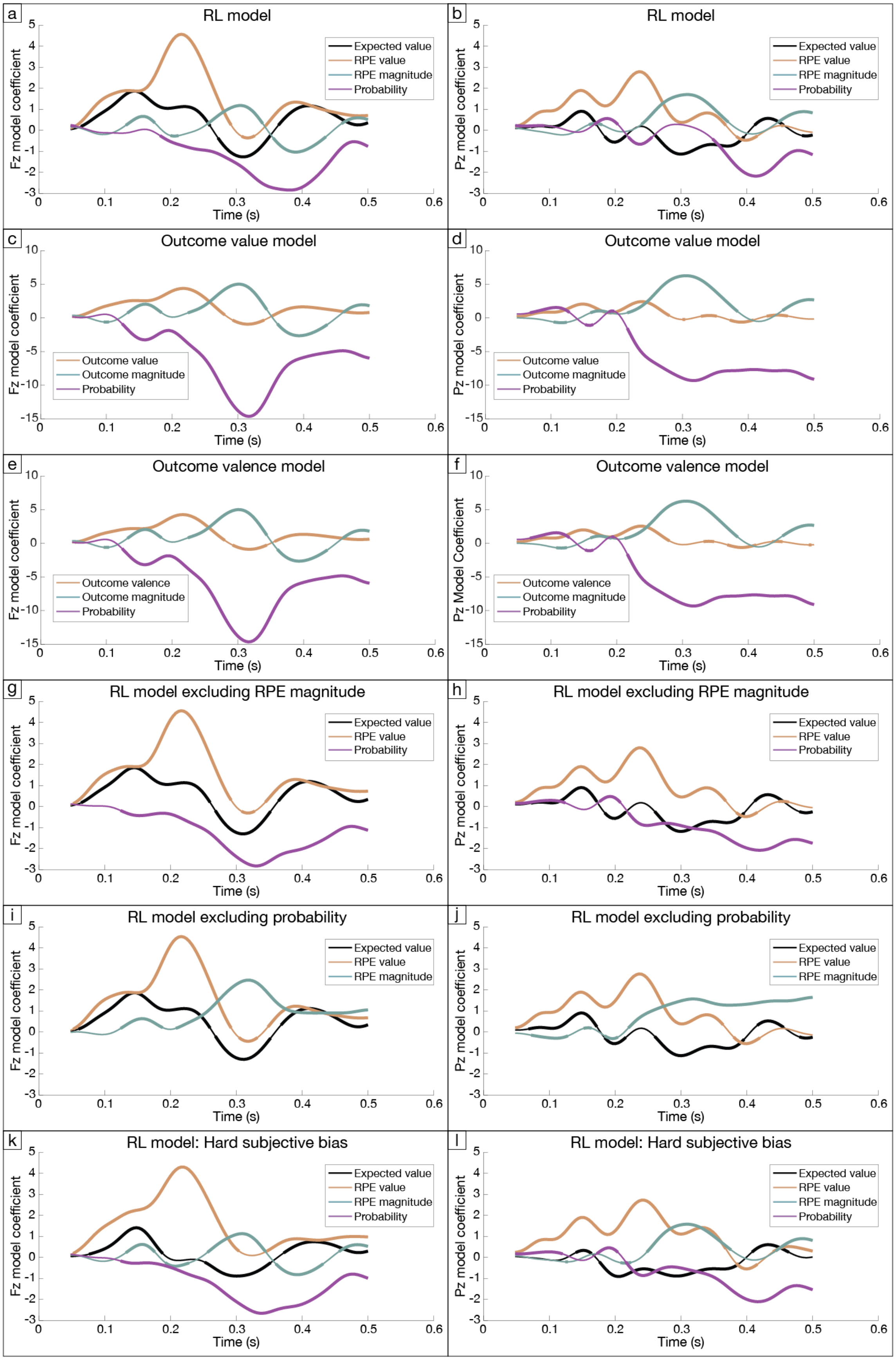
a
RL model
Fz model coefficient
Time (s)
Expected value
RPE value
RPE magnitude
Probability
b
RL model
Pz model coefficient
Time (s)
Expected value
RPE value
RPE magnitude
Probability
c
Outcome value model
Fz model coefficient
Time (s)
Outcome value
Outcome magnitude
Probability
d
Outcome value model
Pz model coefficient
Time (s)
Outcome value
Outcome magnitude
Probability
e
Outcome valence model
Fz model coefficient
Time (s)
Outcome valence
Outcome magnitude
Probability
f
Outcome valence model
Pz Model Coefficient
Time (s)
Outcome valence
Outcome magnitude
Probability
g
RL model excluding RPE magnitude
Fz model coefficient
Time (s)
Expected value
RPE value
Probability
h
RL model excluding RPE magnitude
Pz model coefficient
Time (s)
Expected value
RPE value
Probability
i
RL model excluding probability
Fz model coefficient
Time (s)
Expected value
RPE value
RPE magnitude
j
RL model excluding probability
Pz model coefficient
Time (s)
Expected value
RPE value
RPE magnitude
k
RL model: Hard subjective bias
Fz model coefficient
Time (s)
Expected value
RPE value
RPE magnitude
Probability
l
RL model: Hard subjective bias
Pz model coefficient
Time (s)
Expected value
RPE value
RPE magnitude
Probability

Supplementary Figure 4: Coefficients for alternative and control models. Left column shows results for Fz, and right column shows results for Pz. Bolding indicates significant time points ($q_{FDR} < 0.05$; n = 32 participants). (a) Reference results from best performing RL model at Fz as displayed in Fig. 2c. (b) Same for Pz. (c) Coefficients at Fz for outcome value model show similar results as the RL model but with weaker model performance, especially during the FRN window. (d) Same for Pz. (e) Coefficients at Fz for outcome valence model show similar results as the RL model but with weaker model performance, especially during the FRN window. (f) Same at Pz. (g) Coefficients at Fz for RL model when excluding RPE magnitude, which shows RPE value and probability effects hold but RPE magnitude effect for the P3 is missing. (h) Same for Pz. (i) Coefficients at Fz for RL model when excluding probability, which shows RPE value and RPE magnitude effects hold but late frontal probability effect is missed. (j) Same for Pz. (k) Coefficients at Fz for the RL model after introducing a positive subjective bias on hard trials to match behavioral rating data, which shows the original RL model results are robust against simulated subjective biases. (l) Same for Pz.

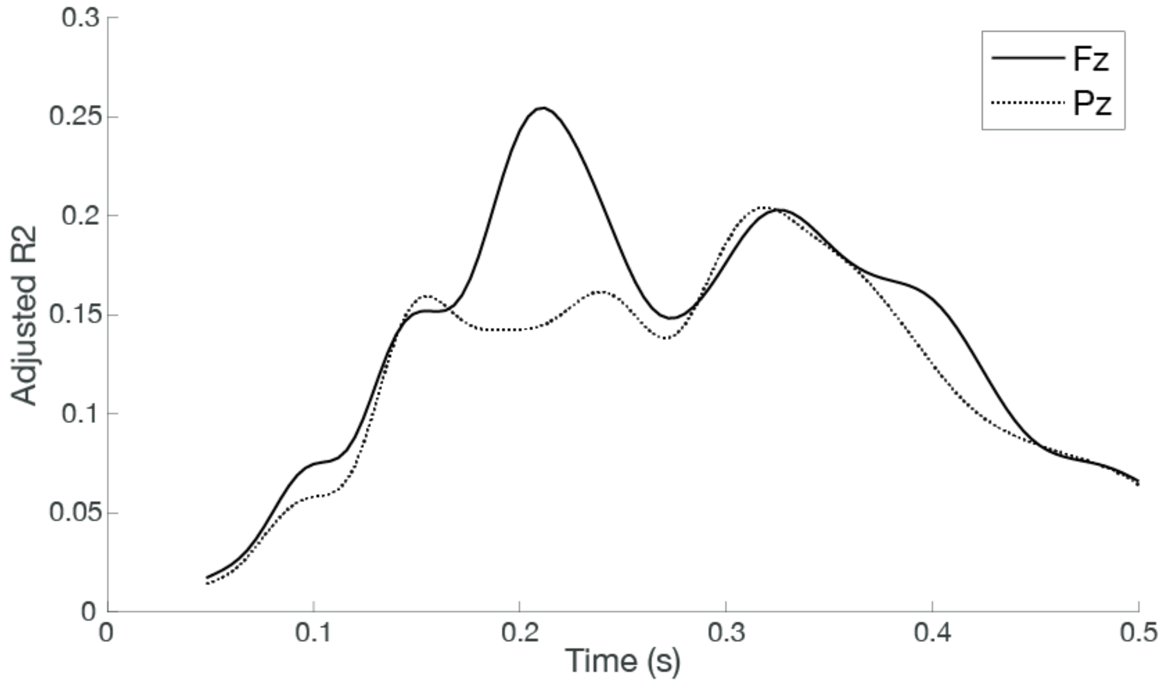

Supplementary Figure 5: ERP amplitude model fits. RL model fits at each time point for frontal electrode Fz and posterior electrode Pz plotted as adjusted R^2. Note that performance peaks in the FRN time window for Fz ($R^2 = 0.254$ at 212 ms) and in the P3 time window for Pz ($R^2 = 0.204$ at 320 ms).

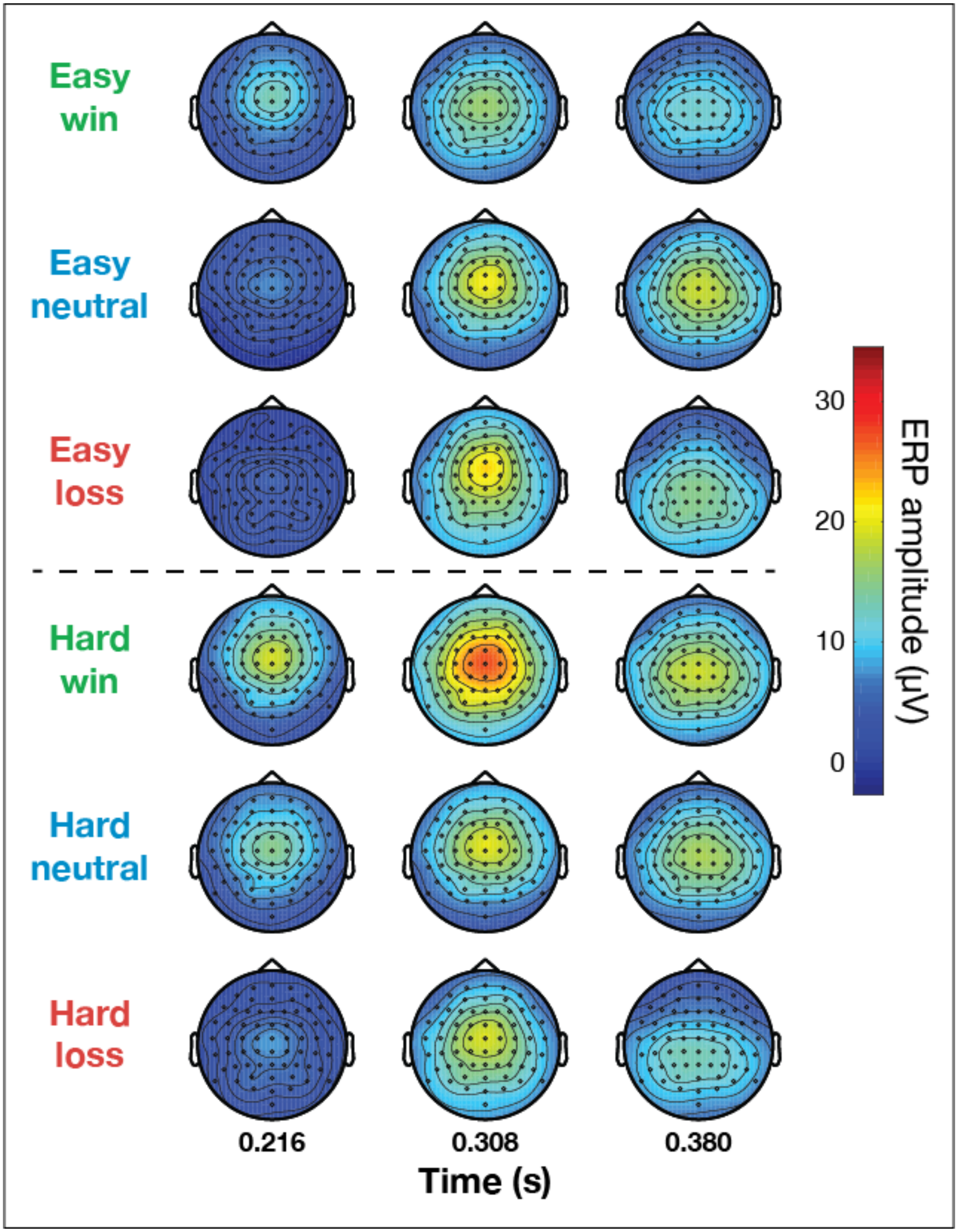

Supplementary Figure 6: ERP amplitude topography dynamics. ERP amplitudes are averaged within condition and participant in 50 ms windows centered on the peak model coefficients for RPE value (0.216 s), RPE magnitude (0.308 s), and probability (0.380 s)

from Fig. 2. Topographies show group averaged amplitude for all electrodes in each window.

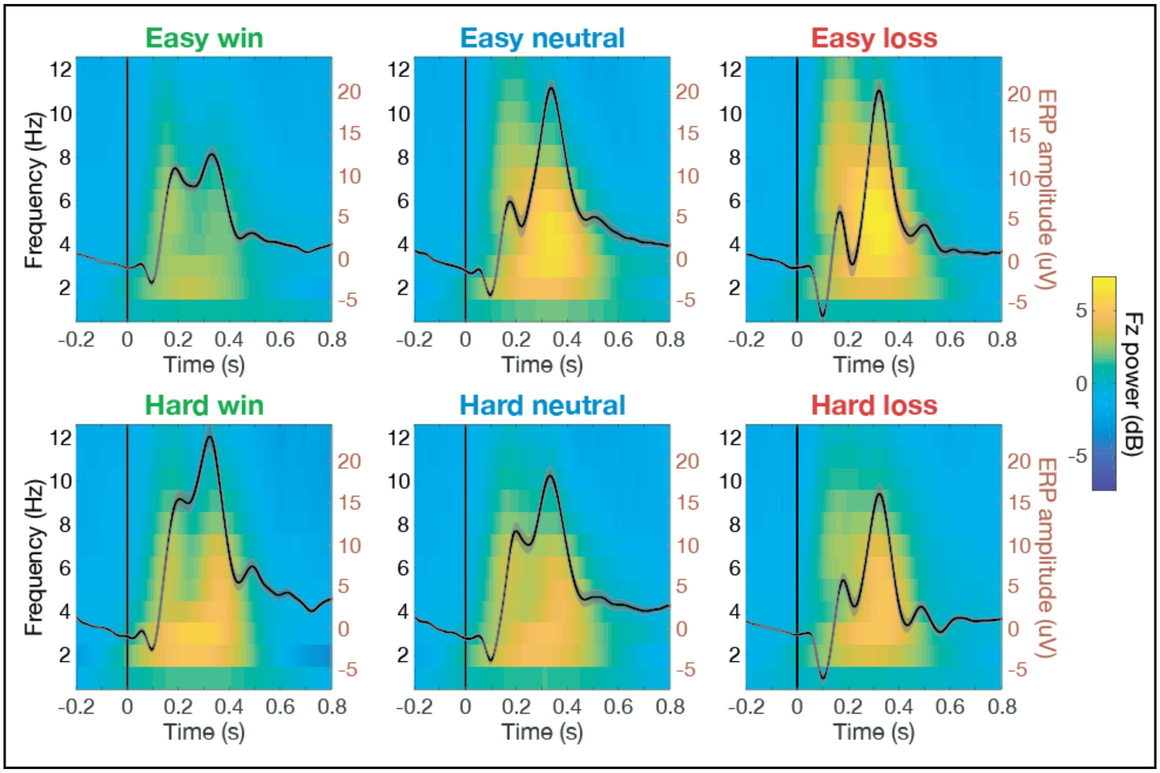

Supplementary Figure 7: Time-frequency evoked power at Fz for each condition. Grand-average ERPs are overlaid on the right y-axis to show the waveshape features driving evoked power, with error bars indicating standard error of the mean.

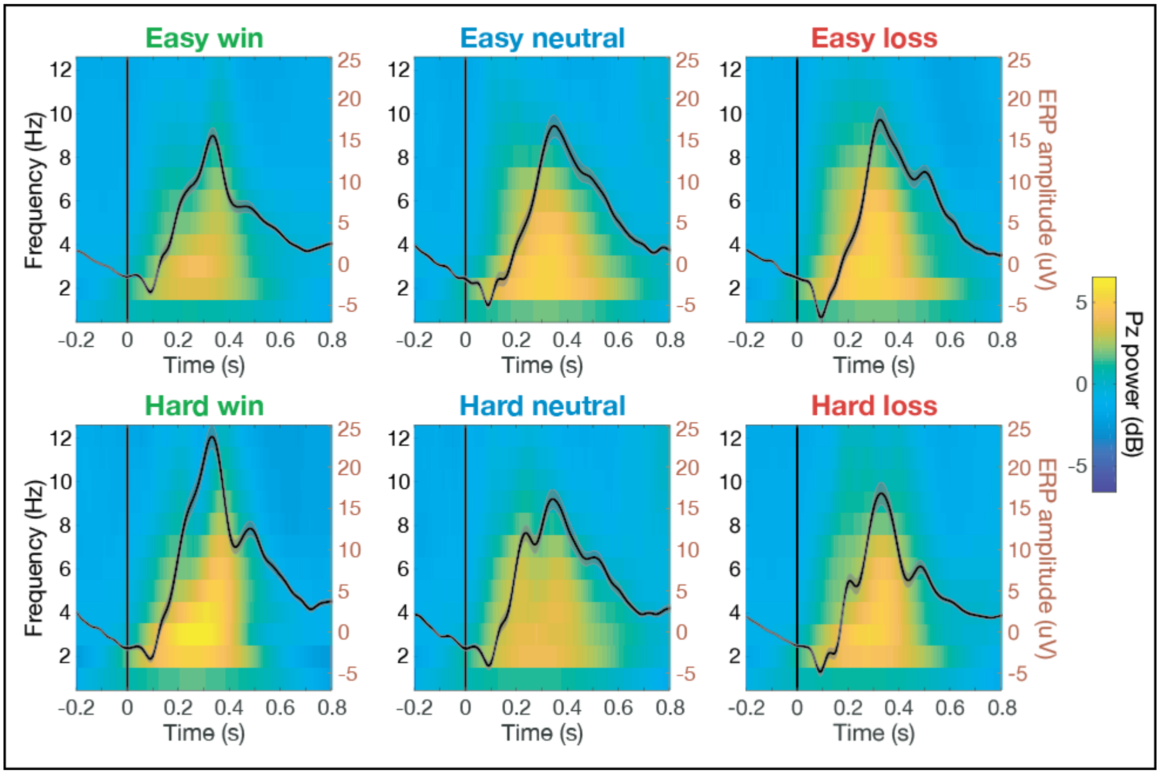

Supplementary Figure 8: Time-frequency evoked power at Pz for each condition. Grand-average ERPs are overlaid on the right y-axis to show the waveshape features driving evoked power, with error bars indicating standard error of the mean.

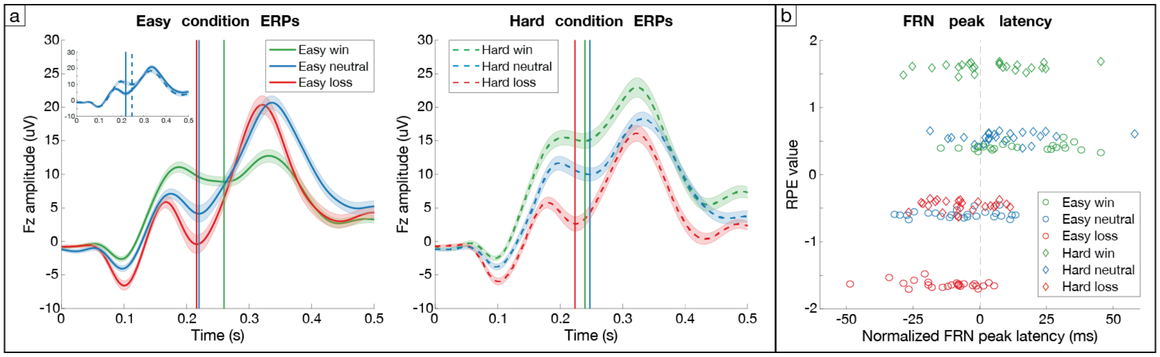

Supplementary Figure 9: FRN peak latency is modulated by valenced RPE value. (a) Grand-average ERPs at Fz for easy and hard conditions separately with the FRN peak

latency marked by vertical lines. Shaded error bars indicate standard error of the mean across participants. Note that peak latencies for identical neutral outcomes shift early for negative RPEs in easy blocks versus late for positive RPEs in hard blocks (see top left inset for direct comparison). (b) Multiple regression revealed RPE value significantly predicted FRN peak latency ($q_{FDR} = 2.80 * 10^{-8}$; n = 32 participants). FRN peak latencies for all participants and conditions are plotted after normalizing for mean FRN latency within participant.

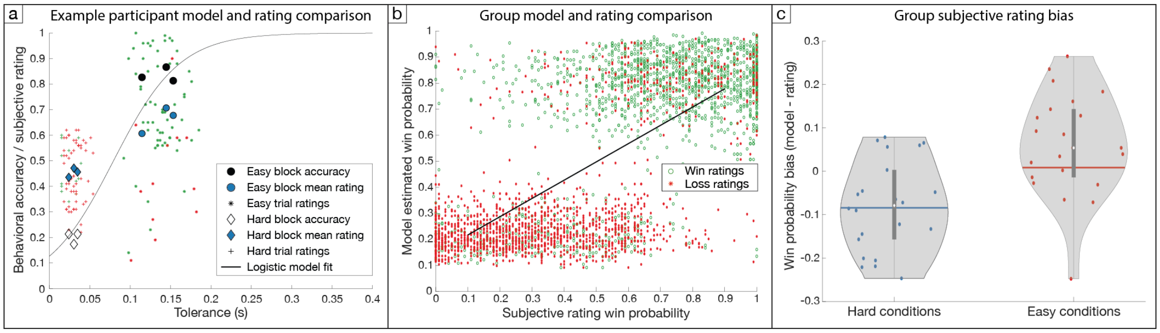

Supplementary Figure 10: Subjective ratings of reward expectations correspond with behavioral modeling but with slight bias. (a) Comparison of win probabilities derived from subjective ratings and logistic model fit to behavioral outcomes in an example participant. Smaller single trial rating markers colored green for correct and red for incorrect responses show overall distinction between easy and hard conditions. Larger markers of block-averaged data show subjective ratings are positively biased in hard conditions and negatively biased in easy conditions relative to objective behavioral accuracy. (b) Group level comparison of single-trial win probability as estimated by subjective ratings and behavioral modeling. Linear fit overlaid as black line for visualization of broad agreement. (c) Group level bias computed as the average

difference between model-based and subjective rating win probabilities confirm overall positive bias in hard conditions and negative bias in easy conditions. Violin plots show kernel density estimate, and individual participant data is plotted as colored points. Group mean (n = 22 participants) plotted as colored horizontal line, with the inter-quartile range plotted as gray vertical bar and the median plotted as a white point.

SUPPLEMENTARY TABLES:

ERP Metric	Cohort	Model Coefficients			
		Expected Value	RPE Value	RPE Magnitude	Probability
FRN Mean Window	1	0.843*	4.237***	0.960*	-0.053
	2	0.454	2.755***	-0.161	-0.859**
	Both	0.602*	3.443***	0.382	-0.508*
FRN Peak-to-Peak	1	-0.371	-1.686*	0.990	0.224
	2	0.413	-0.894*	0.391	-0.041
	Both	0.037	-1.277**	0.666*	0.083
P3 Mean Window	1	-0.822	0.567	1.887**	-0.028
	2	-0.422	0.862*	1.644**	0.208
	Both	-0.599	0.759*	1.721***	0.093

Supplementary Table 1: Reproducibility of model coefficients for traditional ERP metrics across cohorts.

Model coefficients from RL model for linear mixed-effects multiple regression of traditional FRN mean window, FRN peak-to-peak, and P3 mean window ERP metrics are reported for two cohorts separately and combined. RPE value and RPE magnitude results are reproducible for FRN and P3 metrics, respectively, but significance of other predictors varies across cohorts. Stars indicate significance (*: $q_{FDR} < 0.05$; **: $q_{FDR} < 10^{-3}$; ***: $q_{FDR} < 10^{-6}$).

Model	Mean AIC at Frontal Site Fz			Mean AIC at Posterior Site Pz		
	191-241 ms	283-333 ms	355-405 ms	191-241 ms	283-333 ms	355-405 ms
Outcome Value	-1906	-899	-705	-374	-347	-314
Outcome Valence	-2045	-901	-656	-509	-347	-316
Full RL	-2325*	-919*	-721*	-673 (RL = 0.54)	-409*	-336*
RL without RPE Magnitude	-2325 (RL = 0.78)	-862	-680	-674*	-276	-330 (RL = 0.04)

RL without Probability	-2293	-746	-294	-662	-409 (RL = 0.83)	-220
Null Model	142,897	145,788	145,056	141,780	146,384	147,407

Supplementary Table 2: ERP amplitude model performance comparisons.

Model performance is reported as AIC averaged within three windows at Fz and Pz for all outcome-based and RL-based models relative to baseline AIC from a null model including only random effects for participants. Mean AIC for the null model is reported at the bottom. Lower AIC indicates better model performance. Stars indicate the best performing model for a given epoch and electrode. Relative likelihoods are reported for competing models with probabilities exceeding 1%.
